# Predictors for Assessing Electronic Messaging Between Nurses and General Practitioners as a Useful Tool for Communication in Home Health Care Services: A Cross-Sectional Study

**DOI:** 10.2196/jmir.4056

**Published:** 2015-02-17

**Authors:** Merete Lyngstad, Dag Hofoss, Anders Grimsmo, Ragnhild Hellesø

**Affiliations:** ^1^Institute of Health and SocietyDepartment of Nursing ScienceUniversity of OsloOsloNorway; ^2^Department of Public Health and General PracticeNorwegian University of Science and TechnologyTrondheimNorway

**Keywords:** electronic mail, home healthcare nursing, collaborating, general practitioners

## Abstract

**Background:**

Nurses providing home health care services are dependent on access to patient information and communicating with general practitioners (GPs) to deliver safe and effective health care to patients. Information and communication technology (ICT) systems are viewed as powerful tools for this purpose. In Norway, a standardized electronic messaging (e-messaging) system is currently being established in health care.

**Objective:**

The aim of this study was to explore home health care nurses’ assessments of the utility of the e-messaging system for communicating with GPs and identify elements that influence the assessment of e-messaging as a useful communication tool.

**Methods:**

The data were collected using a self-developed questionnaire based on variables identified by focus group interviews with home health care nurses (n=425) who used e-messaging and existing research. Data were analyzed using logistic regression analyses.

**Results:**

Over two-thirds (425/632, 67.2%) of the home health care nurses returned the questionnaire. A high proportion (388/399, 97.2%) of the home health care nurses who returned the questionnaire found the e-messaging system to be a useful tool for communication with GPs. The odds of reporting that e-messaging was a useful tool were over five times higher (OR 5.1, CI 2.489-10.631, *P*<.001) if the nurses agreed or strongly agreed that e-messaging was easy to use. The odds of finding e-messaging easy to use were nearly seven times higher (OR 6.9, CI 1.713-27.899, *P*=.007) if the nurses did not consider the system functionality poor. If the nurses had received training in the use of e-messaging, the odds were over six times higher (OR 6.6, CI 2.515-17.437, *P*<.001) that they would consider e-messaging easy to use. The odds that a home health care nurse would experience e-messaging as easy to use increased as the full-time equivalent percentage of the nurses increased (OR 1.032, CI 1.001-1.064, *P*=.045).

**Conclusions:**

This study has shown that technical (ease of use and system functionality), organizational (training), and individual (full-time equivalent percentage) elements had an impact on home health care nurses’ assessments of using e-messaging to communicate with GPs. By identifying these elements, it is easier to determine which interventions are the most important for the development and implementation of ICT systems in home health care services.

## Introduction

Health care services are characterized as fragmented and dispersed [1,2]. This issue is particularly challenging for the delivery of safe and effective health care to patients who receive home health care services. Nurses in home health care services are dependent on access to accurate and relevant patient information [3]. It is also essential that nurses in home health care services have the opportunity for clinical communication and discussions about patient care with general practitioners (GPs) [2,4].

The current study was conducted in Norway, where home health care service is managed by municipalities; furthermore, GPs are self-employed [5] and each municipality can use different electronic health record (EHR) systems [6]. Thus, new methods for information exchange and clinical communication between GPs and home health care nurses are needed. Information and communication technology (ICT) systems are viewed as powerful tools that can solve this problem [7]. One of the most important goals for ICT development and implementation in health care is to improve the exchange of information, care coordination, and communication between health care workers [7,8]. However, implementing new electronic tools in health care has not always been successful [9,10]. Studies have shown that health care professionals may assess the tools as inefficient or believe that the tools do not align with their work practices [11-13]. Health care professionals’ beliefs, assessments, and satisfaction with the ICT systems influence the structure, process, and outcome of care, which can affect the safety of patients and their next of kin [3,14,15]. Developing and implementing new electronic tools for communication has high costs; therefore, it is important to reduce the risks of low, incorrect, or incomplete use of the systems [9,16].

A sociotechnical perspective aims to understand how ICT systems are developed, implemented, and become a part of standard work practices [17]. The integration of ICT systems into work practices is dependent on the interaction between individual, organizational, and technological elements. These elements determine whether the implementation and use of ICT systems will be successful [16]. Therefore, a technical system cannot be isolated and function on its own and instead needs to be shaped and reshaped by the people and organizations in the social environments and contexts in which it is used [17]. The sociotechnical perspective emphasizes the need for active user involvement in an iterative development and implementation process and considers strong user involvement for adopting ICT systems, and for assessing them as useful [8,15,18-20].

Previous studies have shown that several elements impact health care professionals’ assessments of the usability of ICT systems. These elements include the health professionals’ participation in the development of the ICT system [19], the design of the ICT system [11,12,21], the user interface and functionality of the ICT system [15], the ease of use [20-24], the compatibility with work practices [11,15,21,22,25], and how the ICT systems are put into practice (eg, training, user support, technical infrastructure, and resources) [7,11,15,21,23,26-28].

In Norway, a standardized electronic messaging (e-messaging) system is currently being implemented in primary health services [29]. The e-messaging system has been integrated into all of the major EHR systems used in home health care services and GP offices in Norway, meaning that it is a module of the EHR systems that can be procured from vendors. The e-messaging system consists of six standardized, semistructured message types that can be sent from home health care nurses to GPs and five semistructured message types that can be sent from GPs to home health care nurses. The e-message types are customized for different purposes, including the patients’ health information and medication lists. A dialogue message was designed for requests, inquiries, and discussions about patients’ health issues and special care needs, administrative information on the type of home health care services provided to the patient and information about errors and omission in the treatment and care of the patient [30]. The e-messages are sent between the different health care providers’ EHR systems via the Norwegian Health Net, which is a secure and closed net used solely for health information exchange and clinical communication.

The development of the e-messaging system was initiated and managed by health care professionals in close cooperation with vendors [31]. Several home health care nurses specified the requirements of the e-messaging system and participated in the development and implementation phases of the e-messaging system [31]. Thus, the system was adjusted by strong user involvement as suggested in other studies [15,19,20,22]. The Norwegian government aims to implement e-messaging in home health care services, nursing homes, GP offices, and hospitals by the end of 2014 [6].

The aim of this paper was to describe home health care nurses’ assessments of using the e-messaging system to communicate with GPs and identify which elements predict that e-messaging will be considered a useful tool.

## Methods

The study used a cross-sectional approach with a questionnaire administered between November 2011 and February 2012.

### The Questionnaire

We searched the literature but did not find any questionnaires that measured the use of e-messaging between home health care services and GPs. Therefore, we developed a questionnaire in two phases [32]. In the first phase, we explored the recommended guidelines for the use of e-messages [33], the description of the standards for the e-messaging system [30], and the results from previous contiguous research [34-37]. We then conducted focus groups and semistructured interviews with home health care nurses from two municipalities. The results from the interviews showed that both organizational and technical elements were assessed as important dimensions for the use of e-messaging [38].

The second phase consisted of the questionnaire development. The questionnaire was reviewed by 6 registered nurses with clinical, ICT, and research skills, and it was pilot tested by 15 registered nurses who worked in home health care services. These steps provided significant and valuable information that was used to revise the questionnaire. The final version of the questionnaire contained 62 items in six sections: (1) Demographic information, eight items, (2) Procedures for and amount of information exchange and communication with GPs, 19 items, (3) Information content, 12 items, (4) Expectations for electronic communication with GPs, 10 items, (5) Electronic communication, three items, and (6) Experiences of the use of electronic communication, 10 items (see Multimedia Appendix 1). Six items were negatively worded: Items 19 (a)-(d) and Items 20 (d) and (e). Here, we report the demographic, electronic communication, and experiences of the use of e-messaging results from Sections 1, 5, and 6, respectively.

The items selected for this study have been previously documented as important factors for health care providers’ assessments of health information systems [7,9,11,15,21,26-28]. These items can be grouped as individual, organizational, and technological elements. The individual element consists of Items 2-6 from Section 1 and Item 16 from Section 5 of the questionnaire. The organizational element consists of Items 20 (a) and (b) from Section 6 of the questionnaire. The technological element consists of Items 19 (b)-(d) and 20 (c) from Section 6 of the questionnaire.

The questionnaire items were rated on a 5-point Likert scale as follows: (1) strongly disagree, (2) disagree, (3) neither agree or disagree, (4) agree, and (5) strongly agree. The Cronbach alpha exceeded .7, indicating acceptable reliability [39].

To test the validity of the questionnaire, a selection of the items from the main study was summarized and correlated with the item “useful tool for communication with GPs”, which is the questionnaire’s concluding question. Pearson’s *r* was .57 (*P*<.001), indicating a fairly strong correlation between the overarching concept of usefulness of the e-messaging tool and the selected questionnaire items, but also that there may be items or facets of items that should have been addressed in the questionnaire. However, we wanted to keep the questionnaire short to achieve a high response rate. The pilot test showed that it took approximately 10 minutes to complete the questionnaire.

### Data Collection

The questionnaire data were collected from home health care nurses in 12 out of 428 municipalities in Norway. The inclusion criterion for the municipalities required that the e-messaging system had been in use for more than 6 months. We assumed that system usage would be stable after this time frame [40]. The 12 municipalities were the first in Norway to introduce e-messaging to support communication between home health care nurses and GPs, and they were the only municipalities that met this inclusion criteria at the time of our study. All of the home health care nurses were employed in a 50% or greater full-time equivalent position, had been using e-messaging for at least 3 months, and were able to read and write Norwegian. The home health care nurses received the questionnaire, information letter, and return envelope at their workplace from a designated contact person in each municipality. The contacts were responsible for distributing collective reminders and for collecting the envelopes with the completed questionnaire. There were 681 nurses qualified to answer the questionnaire, and the questionnaire was distributed to 632 (92.8%) nurses.

### Ethical Considerations

The home health care nurses who were invited to answer the questionnaire received written information explaining that their participation was anonymous and that returning the questionnaire meant that they agreed to participate in the study. The methods for data collection and handling the interviews and the questionnaire were approved by the Norwegian Social Science Data Services, which is the official data protection agency for research at the University of Oslo (reference no. 26230).

### Data Analysis

We used a descriptive analysis to determine the characteristics of the sample and the nurses’ assessments of using e-messaging. Scores for negatively worded items were reversed. Two logistic regression models were developed to determine the extent to which specific elements influenced the odds that the nurses would report that e-messaging was a useful and easy tool for communication with GPs.

In the first logistic regression model, “the useful tool model”, we chose to include the independent variables of “easy to use” and the home health care nurses’ demographic characteristics of “age”, “gender”, “full-time equivalent percentages”, “years of experience as a registered nurse (RN)”, “years in current position”, and “number of months using e-messaging”. The reason for choosing these variables is based on results from previous research [3,8,21,26,38].

In the second logistic regression model, “the ease-of-use model”, the dependent variable of “easy to use” was combined with the independent variables of “received training”, “access to user support”, “hindered by poor functionality”, “hindered by low system performance”, and “hindered by software error”, and the home health care nurses’ characteristics. These variables have been shown to have an impact of the assessment and adoption of ICT systems [3,7-9,11,15,21,26-28,38].

In the pre-analysis of the data, we searched for outliers in continuous variables. The results showed that it was not necessary to transform any of the data. We used ±3.30 standard deviations (SD) to check for outliers, as suggested by Altman [41]. Two variables had outliers: “years in current position” with 24 years (0.9%) in the positive direction and “number of months using e-messaging” with 37 months (1.3%). However, working for an extended period of time in the same position or using the e-messaging system for 37 months is unlikely to affect the results.

Homoscedasticity in both logistic regression models was assessed by a one-way analysis of variance of the standardized residuals to establish that their variance was approximately the same for all values of the predictor variables. No homoscedasticity was found. The first model was used to predict if the e-messaging system was a useful tool for communication with GPs. The item “easy to use” did not show statistically significant differences (*P*=.76) between the prediction errors in the group that found the e-messaging system to be a useful tool and the group that did not find the tool to be useful. The second model was used to predict if the e-messaging system was easy to use. The items “received training” (*P*=.88), “hindered by poor functionality” (*P*=.84), and “full-time equivalent percentage” (*P*=.77) did not show statistically significant differences between the prediction errors in the group that found the e-messaging system easy to use and the group that did not find the tool easy to use.

We tested for possible multicollinearity among the independent variables in both models using the Pearson correlation to exclude that the independent variables were highly correlated. The highest correlation was .76 for age and years of experience as an RN; therefore, no significant multicollinearity was found [42].

In the logistic regression analysis, we dichotomized the variables because of their skewed distribution. The variable “useful tool” was dichotomized as strongly disagree, disagree, neutral, and agree (0=1-4) and strongly agree (1=5). The rest of the ordinal variables were dichotomized as strongly disagree, disagree, neutral (0=1-3), agree, and strongly agree (1=4-5).

The *P* value of the Hosmer and Lemeshow goodness of fit statistic for “the useful tool model” was .317, and for “the ease-of-use model” the *P* value was .650. The data were analyzed using IBM SPSS Statistics version 20.0.

## Results

### Characteristics of Home Health Care Nurses

A total of 425 (67.2%) of the 632 home health care nurses who received the questionnaire completed it. The demographic information is presented in Table 1.

**Table 1:** Demographic information of home health care nurses (N=425).

Demographic characteristics		Mean	n (%)
**Gender, n (%)**			
	Female		383 (90.4)
	Male		41 (9.6)
Age, mean (SD)		39.6 (10.1)	424 (99.8)
Years of experience, mean (SD)		11.5 (9.1)	421 (99.1)
Full-time equivalent percentage, mean (SD)		90.9 (14.2)	425 (100.0)
Years in current position, mean (SD)		5.8 (5.6)	422 (99.3)
Number of months using e-messaging, mean (SD)		10.21 (7.5)	382 (89.9)

### Home Health Care Nurses’ Assessments of e-Messaging

The vast majority of the responding home health care nurses (388/399, 97.2%) agreed or strongly agreed that e-messaging was a useful tool for communication with GPs. Table 2 presents all responses in order to show the complete distribution of the responses.

For the “easy to use e-messaging” item, the majority of home health care nurses agreed or strongly agreed (357/398, 89.7%) that e-messaging was easy to use. A high proportion of the home health care nurses agreed or strongly agreed that they had received training (333/399, 83.5%), while the remaining nurses were neutral or disagreed. The home health care nurses agreed to a lower degree that they had access to user support (256/389, 65.8%).

A relatively high proportion of the home health care nurses were neutral or agreed that they were hindered when using e-messaging because of poor functionality (123/395, 31.1%), low system performance (123/396, 31.1%), or software errors (129/395, 32.7%).

**Table 2:** Assessments of using e-messaging (N=425).

Item	Strongly disagree, % (n)	Disagree, % (n)	Neutral, % (n)	Agree, % (n)	Strongly agree, % (n)	Missing, n
Received training	1.7 (7)	6.0 (24)	8.8 (35)	50.9 (203)	32.6 (130)	26
Access to user support	1.2 (5)	3.6 (14)	29.3(114)	52.9 (206)	12.9 (50)	36
Hindered by poor functionality	18.7 (74)	50.1 (198)	23.3 (92)	6.6 (26)	1.2 (5)	30
Hindered by low system performance	19.4 (77)	49.5 (196)	23.5 (93)	6.3 (25)	1.2 (5)	29
Hindered by software error	21.5 (85)	45.8 (181)	17.2 (68)	13.1 (52)	2.3 (9)	30
Easy to use	0.2 (1)	1.5 (6)	8.5 (34)	52.8 (210)	36.9 (147)	27
Useful tool	-	0.7 (3)	2 (8)	31.8 (127)	65.4 (261)	26

### Predictors That Affected Nurses’ Assessment of e-Messaging as a Useful Tool for Communication With GPs

The logistic regression model showed that the “easy to use” item gave a statistically significant contribution to the model (Table 3).

The odds of reporting that e-messaging was a useful tool were over five times higher if the nurses agreed or strongly agreed that e-messaging was easy to use. The “useful tool model” was controlled for the demographic variables of gender, age, years of experience as an RN, full-time equivalent percentage, and years in current position.

**Table 3:** Logistic regression model examining predictors affecting the assessment of e-messaging as a useful tool (N=391).

	B (SE)	*P* value	Odds ratio (95% CI)
Gender	.041 (0.402)	.920	1.041 (0.473-2.291)
Age	.009 (0.018)	.621	1.009 (0.974-1.045)
Years of experience as an RN	-.014 (0.021)	.505	0.986 (0.948-1.027)
Full-time equivalent percentage	.006 (0.008)	.505	1.006 (0.989-1.022)
Years in current position	.001 (0.024)	.973	1.1001 (0.955-1.049)
Easy to use	1.638 (0.370)	.000	5.144 (2.489-10.631)
Constant	-1.555	.134	0.211

### Predictors That Affected the Nurses’ Assessment of e-Messaging as Easy to Use

The “ease-of-use model” determined which elements influenced the assessment of e-messaging as easy to use. The results from the logistic regression model (Table 4) showed that the independent variables “hindered by poor functionality”, “training received”, and “full-time equivalent percentage” were statistically significant.

The odds that a home health care nurse would experience e-messaging as easy to use increased as the full-time equivalent percentage of the nurses increased. If the home health care nurses agreed that they were not hindered by poor e-messaging functionality, the odds of agreeing that e-messaging was easy to use were over 6.9 times higher than if they did not agree. The home health care nurses who agreed that they had received training had odds over 6.6 times higher of agreeing that e-messaging was easy to use. The home health care nurses’ gender, number of months using e-messaging, being hindered by low system performance or software errors, or having access to user support were not statistically significant predictors for the assessment of e-messaging as easy to use.

**Table 4:** Logistic regression model examining predictors affecting assessments of e-messaging as easy to use (N=364).

	B (SE)	*P* value	Odds ratio (95% CI)
Gender	-.561 (0.796)	.480	0.570 (0.120-2.713)
Age	-.049 (0.038)	.193	0.952 (0.884-1.025)
Years of experience	-.015 (0.039)	.701	0.985 (0.913-1.063)
Full-time equivalent percentage	.031 (0.016)	.045	1.032 (1.001-1.064)
Years in current position	-.030 (0.043)	.488	0.971 (0.893-1.056)
Number of months using e-messaging	-.030 (0.030)	.318	0.971 (0.915-1.029)
Hindered by low system performance	-.633 (0.731)	.387	0.531 (0.127-2.226)
Hindered by software error	-.094 (0.548)	.863	0.910 (0.311-2.664)
Hindered by poor functionality	1.933 (0.712)	.007	6.914 (1.713-27.899)
Received training	1.890 (0.494)	<.001	6.622 (2.515-17.437)
Having access to user support	.170 (0.479)	.722	1.186 (.464-3.031)
Constant	1.053 (2.001)	.599	2.867

## Discussion

### Principal Findings

The results from this study showed that individual, organizational, and technological elements are interrelated and affect home health care nurses’ assessments of using e-messaging to communicate with GPs. This is in line with a sociotechnical perspective [20]. This study demonstrated that the majority of the home health care nurses assessed the e-messaging system as a useful tool for communication with GPs.

Several home health care nurses cooperated with the vendors in the development and implementation of the e-messaging system. The realization of an ICT system relies on the participation of the people who will ultimately use it [19]. This is important because the system must fit the needs and working practices of the users. ICT development and implementation projects in health care can be controversial because ICT systems change organizational routines and relationships between different health care professionals. To prevent resistance and non-utilization of the ICT systems, users need to be thoroughly and systematically involved at an early stage in the development and implementation process [20]. The involvement of home health care nurses in the development and implementation of the e-messaging system may have resulted in a system that was better aligned with nurses’ needs and working practices; therefore, a positive assessment of the e-messaging system could be anticipated.

The only statistically significant predictor of e-messaging as a useful tool for communication with GPs was that nurses assessed e-messaging as easy to use. This is supported by a study of health care professionals’ adoption and use of a clinical information system that found that the ease of use of the system was required for it to be considered as a beneficial tool for their clinical practice [24].

Ease of use is one of the most frequent elements reported among studies of facilitators and barriers of the adoption of ICT systems in health care [21,22,24]. Ease of use is related to individual characteristics of the people who are using ICT systems, technical features of the ICT system such as the software and hardware, and organizational implementation of the ICT system in terms of training, procedures, user support, and configuration of the system. We found that only one individual element, full-time equivalent percentage, was a statistically significant predictor for e-messaging being easy to use. Previous research has reported opposing evidence that multiple individual elements, such as age, gender, years of experience as a nurse, and full-time or part-time work, affect the assessment of ICT systems [3,8,43-45]. However, we found that the higher the full-time equivalent percentage of the home health care nurses, the more likely they were to assess e-messaging as easy to use. One explanation for this finding is that the more time home health care nurses spend at work, the more they have access to training, user support, and help from their colleagues; thus, the nurse is more familiar with the use of e-messaging, which may result in a positive experience and assessment of the e-messaging system. Another explanation could be that the more time spent at work, the more time is spent using the system, and the more adept the user becomes with the system.

Tools/technical concerns, such as functionality and system design, are important elements that can act as barriers and facilitators for the implementation and use of ICT systems in health care [28]. In our study, a lack of poor functionality was the strongest predictor for the home health care nurses’ assessments of e-messaging as easy to use. This finding is supported by another study on nurses’ assessments of health care technology, which revealed that poor system design was among the most common elements for negative attitudes [12]. Ease of use is also related to the technical environment and the integration between ICT systems. In a study on usability, the results showed that physicians rated ICT systems as low because the integration between the ICT systems was insufficient [11]. In this study, the technological environment was important in the way that the e-messaging system was integrated into the EHR systems that home health care nurses were already using. The user interface was well known, which lowered the threshold for implementing and adopting the e-messaging system [16].

Assessments of ICT systems are affected by organizational aspects, such as offering user support and training [11]. Our results show that a high proportion of the home health care nurses had received initial training in the use of the e-messaging system, which was a strong predictor for assessing the e-messaging system as easy to use. Training has previously been identified as a key element for the successful implementation of ICT systems in complex systems such as health care services [7,9,15,46,47]. Training is important in ensuring that the systems are used in the intended way and reducing the risk of incorrect use, which could jeopardize patient safety [7,15,27]. Nurses’ use of ICT systems is affected by training in both basic ICT and specific software [3,28,48]. Training at different levels should be offered by organizations and must be tailored to the individual needs of home health care nurses [49].

By applying a sociotechnical perspective, we were able to detect and predict which elements were important for the development and implementation of e-messaging. All of these elements are interrelated and shape the sociotechnical system; furthermore, these elements should be considered when developing and implementing new technology in home health care services [20].

### Limitations

This study has several limitations. The best methodology to develop a questionnaire is to include a pilot study with a smaller sample using all possible variables and subsequently identifying the relevant ones. However, we did not complete this type of study because of our limited time frame. As an alternative, we based the questionnaire on explanatory variables identified by the focus group interviews and existing research.

The questionnaire was not assessed across all dimensions of validity and reliability. However, the aim of the study did not include a complete psychometric testing of the questionnaire.

The mean full-time equivalent percentage may have been falsely high because one of the inclusion criteria for participation in the study was that the nurses had to be engaged in a 50% or greater full-time equivalent position. This was necessary to ensure that the nurses had enough experience in using e-messaging. According to the Norwegian Nurses Organization in 2011, 56% of the nurses in primary care in Norway did not work in a full-time positon, and 15% of those had less than a 50% full-time equivalent position [50]. The odds ratio of the full-time equivalent percentage predictor may be higher than our results suggest; thus, the results in our study may understate the odds of the full-time equivalent predictor in the general population. Another element that may have biased the results was that the home health care nurses who participated in this study worked in the first municipalities in Norway to implement e-messaging. Being among the early adopters may have caused the home health care nurses to be especially encouraged, enthusiastic, technologically optimistic, and positive toward e-messaging. These characteristics could differ in populations that were late to adopt the system.

The strength of this study is that we questioned home health care nurses in all of the municipalities that had used the e-messaging system for at least 6 months at the time of the study. The response rate of the study was 67.2%, which can be considered fairly good for a questionnaire study [51].

### Conclusions

By identifying elements that affect home health care nurses’ assessments of e-messaging for communication with GPs, we are better able to determine which interventions are most important for the development and implementation of e-messaging. This study demonstrated that home health care nurses assessed e-messaging as a useful tool for communication with GPs. It also shows that ICT systems must be easy to use to be assessed as a useful tool and that a higher full-time equivalent percentage, having received training, and not being hindered by poor functionality are important predictors for the assessment of the e-messaging system as easy to use. Our results imply that users should be actively involved in the development and implementation of ICT systems. Future studies should use a sociotechnical approach to consideration the complete range of elements that can affect working practices and the outcomes for the patients and organizations involved. These insights may help to increase the understanding of effective strategies for developing and implementing ICT systems in home health care services.

## Multimedia Appendix 1

The questionnaire.

## Abbreviations

GP: general practitioner
ICT: information and communication technology
RN: registered nurse
