# Supplementary material for: Predictors for Assessing Electronic Messaging Between Nurses and General Practitioners as a Useful Tool for Communication in Home Health Care Services: A Cross-Sectional Study
Source: J Med Internet Res. 2015 Feb 17;17(2):e47. doi: 10.2196/jmir.4056 (PMC4376142; doi:10.2196/jmir.4056)
Supplement: Supplementary file 1 [file jmir_v17i2e47_app1.pdf]

To Nurses:

Participation in a research project: Bridging the Information Gap in Patient Transition (BIG)

This is an invitation to you to participate in the research project BIG. The purpose of BIG is 1) to examine how the introduction of an electronic messaging system influences information exchange and communication between home health care nurses and general practitioners, 2) to examine how the electronic messaging system influence the quality of healthcare to patients that receive community healthcare services. The research project is carried out by a research group at the Institute for Health and Society, University of Oslo, and is financed by the Research Council of Norway (project 196365). The projects supervisors are Associate Professor Ragnhild Hellesø (UiO), Professor Dag Hofoss (UiO) and Professor Anders Grimsmo (NTNU).

We hope that you can use 10 minutes to answer this questionnaire, and returning it in the enclosed envelope. It is voluntary to participate in this study; if you choose not to participate you do not need to provide a reason for this. The completed questionnaire is regarded as an informed consent. The provided information will be treated in such a way that it is not traceable to your person. The University of Oslo is responsible for the use of your information. The project has been assessed by the Data Protection Official for Research, the Norwegian Social Science Data Services, and satisfies the requirements in the Privacy Act. The results from the survey will be used in presentations and publications.

Questions number 1 to 15 pertain to the exchange of information and should be answered by all, independent of if you use or not use electronic messages in your communication with general practitioners. Questions 16 to 20 pertain to the use of the electronic messaging system, and are to be answered only if you use electronic messages. There are different terms that are used when referring to electronic messages, such as e-link, e-messages, care messages. In this questionnaire the term electronic messages is used.

If you have any questions, please contact:
PhD Candidate Merete Lyngstad, telephone 92625044 or email: Merete.Lyngstad@medisin.uio.no
or
Project Manager and Associate Professor Ragnhild Hellesø, telephone 22850566 or email: Ragnhild.Hellseø@medisin.uio.no

Please use a black or blue pen when you fill out the questionnaire, since it provides machine readability. Place the completed questionnaire in the provided envelope and give it to the allocated contact person in the municipality.

Best regards

Merete Lyngstad	Ragnhild Hellesø
PHD Candidate	Associate Professor

©Merete Lyngstad

T T

Demographic information *(please tick and write in the boxes)*

1 What municipality do you work in? ______________________________

2 Are you female ☐ male ☐

3 How old are you? ☐☐ years old

4 How long have you worked as a nurse? ☐☐ years

5 What percentage of a full-time equivalent (FTE) is your position? ☐☐ % of FTE

6 How many years have you been in your current position? ☐☐ years

7 How many patients are there in your zone/department/group? ☐☐ (approx. no.)

8 How many employees are there in your zone/department/group? ☐☐ (approx. no.)

Procedures for and amount of information exchange and communication with GPs

9 I find that ....
(Please answer all the sub-questions)

		Strongly disagree	Disagree	Neither agree or disagree	Agree	Strongly agree
a	there are sound guidelines for exchanging patient information with the GPs	☐	☐	☐	☐	☐
b	it is easy to get in contact with GPs	☐	☐	☐	☐	☐
c	we have regular nurses who are responsible for contacting the GPs..	☐	☐	☐	☐	☐
d	we are maintaining contact with the GPs when the regular nurses are absent	☐	☐	☐	☐	☐

10 I find that ....
(Please answer all the sub-questions)

		Always	Often	Sometimes	Rarely	Never
a	I give information to the GP before a patient encounter.	☐	☐	☐	☐	☐
b	I receive information from the GP after an encounter ...	☐	☐	☐	☐	☐
c	I receive information from the GP if there are any changes in the patient's condition or situation	☐	☐	☐	☐	☐

©Merete Lyngstad

T T

11 Roughly how many times in the last 14 days have you received information or enquiries about patients from GPs using the following methods? *(Please answer all the sub-questions)*

12 Roughly how many times in the last 14 days have you given information or enquiries about patients to GPs using the following methods? *(Please answer all the sub-questions)*

		11 Approx. no. of times	12 Approx. no. of times
a	Telephone…………………..	☐☐	☐☐
b	Letter …………………………	☐☐	☐☐
c	Letter/note delivered via the patient/next of kin.................	☐☐	☐☐
d	Meeting	☐☐	☐☐
e	Verbal message via patient/next of kin	☐☐	☐☐
f	Fax ……………………………	☐☐	☐☐

Information content

13	**I feel that it is important to receive the following information from the GP in order to provide good care to a patient** *(Please answer all the sub-questions)*	Strongly disagree	Disagree	Neither agree or disagree	Agree	Strongly agree
a	Medication………… ……………………………………	☐	☐	☐	☐	☐
b	Intolerances/Allergies…………..………………………..	☐	☐	☐	☐	☐
c	Medical diagnoses	☐	☐	☐	☐	☐
d	What treatment and monitoring the patient is receiving..	☐	☐	☐	☐	☐
e	The patient's level of function.......................................	☐	☐	☐	☐	☐
f	Information given to the patient/family …………………	☐	☐	☐	☐	☐

14	**I usually have up-to-date information in the patient's health records about……** *(Please answer all the sub-questions)*	Strongly disagree	Disagree	Neither agree or disagree	Agree	Strongly agree
a	Medication ………………………………………………	☐	☐	☐	☐	☐
b	Intolerances/Allergies ……………………………………	☐	☐	☐	☐	☐
c	The patient's problems and needs.. ……………………	☐	☐	☐	☐	☐
d	What treatment and monitoring the patient is receiving.	☐	☐	☐	☐	☐
e	The patient's level of function	☐	☐	☐	☐	☐
f	Information given to the patient/family …………………	☐	☐	☐	☐	☐

©Merete Lyngstad

Expectations for electronic communication with GPs

15 I think that the electronic communication between home healthcare and GPs leads to
(Please answer all the sub-questions)

		Strongly disagree	Disagree	Neither agree or disagree	Agree	Strongly agree
**a**	Fewer medication errors	☐	☐	☐	☐	☐
**b**	Fewer errors related to treatment and care	☐	☐	☐	☐	☐
**c**	Treatment and interventions starting quicker	☐	☐	☐	☐	☐
**d**	Better knowledge of the patient's problems and needs……	☐	☐	☐	☐	☐
**e**	More direct patient time	☐	☐	☐	☐	☐
**f**	Better documented health care ………………………………	☐	☐	☐	☐	☐
**g**	More up-to-date information in the patients' health record...	☐	☐	☐	☐	☐
**h**	Spending less time trying to contact the GP	☐	☐	☐	☐	☐
**i**	Easier collaboration with GPs	☐	☐	☐	☐	☐
**j**	An increase in the number of enquiries from GPs	☐	☐	☐	☐	☐

Electronic communication

*(Answer questions 16-20 **only** if you have used electronic messages for communicating with GPs)*

16 How long have you communicated electronically with GPs? ☐☐ months

.

17 If you are expecting an answer to an electronic message from a GP, when do you usually get a reply? *(Please select one option)*

a The same day …………...................................... ☐
b After 1-2 working days …………….............. ☐
c After 3 working days or more .... ☐

18 If you do not get a response from the GP within 3 working days, what do you do? *(You can tick more than one option)*

a Call ……………….. ☐
b Send a new electronic message ………………... ☐
c Go to the GPs office ……………………………. ☐
d Send a letter in the mail .... ☐
e Send a fax ………………………………………… ☐
f Contact the emergency service......................... ☐

©Merete Lyngstad

Experiences of the use of electronic communication

19	**I find that I am prevented from or delayed in using electronic messages** *(Please answer all the sub-questions)*	Strongly disagree	Disagree	Neither agree or disagree	Agree	Strongly agree
a	because the computers are being used by others	☐	☐	☐	☐	☐
b	because of computer errors, crashes or other computer-related problems	☐	☐	☐	☐	☐
c	because the electronic health record system works too slowly ….	☐	☐	☐	☐	☐
d	because of the poor functionality of the electronic health record system......	☐	☐	☐	☐	☐

20	**I find that ...** *(Please answer all the sub-questions)*	Strongly disagree	Disagree	Neither agree or disagree	Agree	Strongly agree
a	I have access to user support	☐	☐	☐	☐	☐
b	I have received training in the use of electronic messages……………………………………………….	☐	☐	☐	☐	☐
c	it is easy to use electronic messages	☐	☐	☐	☐	☐
d	there are too many types of electronic messages	☐	☐	☐	☐	☐
e	it is difficult to decide which message type to use….	☐	☐	☐	☐	☐
f	electronic messages are a useful tool for the exchange of information with GPs	☐	☐	☐	☐	☐

Thank you for taking the time to answer this survey!

©Merete Lyngstad
